# Growth and survival of microencapsulated probiotics prepared by emulsion and internal gelation

**DOI:** 10.1007/s13197-019-03616-w

**Published:** 2019-02-21

**Authors:** Wentao Qi, Xinxiao Liang, Tingting Yun, Weiqun Guo

**Affiliations:** Cereals and Oils Nutrition Research Group, Academy of State Administration of Grain, No. 11 Baiwanzhuang Street, Beijing, 100037 People’s Republic of China

**Keywords:** Microencapsulation, Probiotics, Emulsion and internal gelation, Bacterial growth, Survival rate

## Abstract

Efficient microencapsulation of probiotics by most existing methods is limited by low throughput. In this work, *Saccharomyces boulardii* and *Enterococcus faecium* were microencapsulated by a method based on emulsion and internal gelation. The growth and survival of microencapsulated microbes under different stressors were investigated using free non-encapsulated ones as a control. The results showed that the prepared micro-beads by emulsion and internal gelation exhibited a spherical and smooth shape, with sizes between 300 and 500 μm. Both *S. boulardii* and *E. faecium* grew well and survived better when encapsulated in micro-beads. The survival rates were increased 25% and 40% for microencapsulated *S. boulardii* and *E. faecium* respectively when compared with non-encapsulated controls under high temperature and high humidity. The increases of survival rates were 60% for microencapsulated *S. boulardii* and 25% for *E. faecium* in simulated gastric juice. And the increases were 15% and 20% respectively when the survival rates of the microencapsulated *S. boulardii* and *E. faecium* were determined in simulated intestinal juice. The microencapsulation by emulsion and internal gelation offers an effective way to protect microbes in adverse in vitro and in vivo conditions and is promising for the large-scale production of probiotics microencapsulation.

## Introduction

Probiotics are live microbes that when administered in adequate amounts confer a health benefit (Hill et al. 2014). Nowadays, besides the basic role of nutrition consisting for growth and development of the organism, some additional aspects are becoming increasingly important for counteracting diseases. The quality of food is becoming more and more important because of the problem of food poisoning and nutrition-related chronic disease such as obesity, allergy, cardiovascular diseases, and cancer (John et al. 2011). Many reports point to the health benefits of using probiotics in human nutrition by increasing the body’s immunity (Paulina and Katarzyna 2017; Vandenplas et al. 2013; Ashraf and Shah 2014). Recommendations for probiotics as functional foods or even medicine have been available in several fields (Paulina and Katarzyna 2017; Gill et al. 2015; Hempel et al. 2011).

To achieve the most beneficial effects, probiotics must survive through the gastrointestinal tract and retain bioactivity at their target site. However, several probiotics are incapable of delivering their targeted beneficial effect to the host for many reasons—for example, due to the presence of gastric acids and intestinal juices (Rokka and Rantamäki 2010). Additionally, adverse conditions such as high temperature, high humidity, and drying may elicit a sub-lethal effect on microorganisms (Saarela et al. 2000). Several methods have been used to enhance the viability of probiotics, including selection of acid- and bile-resistant strains, two-step fermentation, stress adaption, added micronutrients, and microencapsulation (Gismondo et al. 1999); of these, microencapsulation has been suggested as an effective method (Anal and Harjinder 2007; Gerez et al. 2012; Arihara 2006). Various materials, such as vegetable gum, starch, cellulose, alginate, and chitosan, have been used as an encapsulating matrix. Alginate is the most commonly used one due to its good film-forming ability, biocompatibility, and controlled-release property (Mattila et al. 2002). Microcapsules made of alginate polymers have been reported can increase the survival of probiotic bacteria in acids, bile, heat, and storage conditions (Lee and Heo 2000; Ding and Shah 2007).

Several methods have been used to produce microcapsules, including extrusion, phase separation (emulsion), and high-voltage electrostatics (Krasaekoopt et al. 2003). The large diameter and bad shape of alginate beads made by extrusion is an insurmountable problem, as it is known that smaller-diameter beads are often favored due to their enhanced rates of bioconversion (Poncelet et al. 1995). The low yield of high voltage electrostatics is unsuitable for industrial scale-up. As an alternative, an emulsification/internal gelation method was studied to produce small-diameter alginate beads on a large scale, with simple reactive conditions, equipment, and operation. This method has been used for cell (Hoesli et al. 2011), DNA (Vaithilingam et al. 2011), and hormone (Guan et al. 2011) entrapment. In this study, two microbial cells widely used in the food industry, *Saccharomyces boulardii* and *Enterococcus faecium*, were firstly microencapsulated in alginate beads by emulsion and internal gelation, and the survivability of the microencapsulated probiotics in adverse in vitro and in vivo conditions was evaluated.

## Materials and methods

### Bacterial strains

Two tested probiotic strains, including *S. boulardii* (CGMCC No. 10381) (Zhang et al. 2015a, b) and *E. faecium* (CGMCC No. 2516) (Zhang et al. 2015a, b), were cultured under microencapsulated and free non-encapsulated conditions. *S*. *boulardii* was grown in YPD broth at 30 °C under aerobic conditions for 14 h. *E. faecium* was grown in MRS broth at 37 °C under anaerobic conditions for 18 h.

### Microencapsulation of probiotics

The microencapsulation of probiotics was performed by a modification of the emulsion method based on references (Hoesli et al. 2011; Guan et al. 2011). Briefly, 100 mL of sterile 1.8% (w/v) sodium alginate and 5 mL of washed and concentrated probiotic bacteria (10^8^ cfu mL^−1^) were mixed with 0.45 g calcium carbonate dissolved in 300 μL sterile water. After homogenization, the mixture was dispersed into a paraffin oil phase that contained 0.2% (w/v) Span80 and was emulsified for 5 min by stirring at 400 rpm. Then, 900 μL of glacial acetic acid dissolved in 10 mL paraffin oil was added, and stirring was continued for 10 min. Clean and sterilized water (100 mL) was added to the emulsification system to draw the micro-beads into the water phase. The oil layer on the top phase was harvested by aspiration, and centrifuged for the next use. After washing with sterile water three times, the microencapsulated bacteria were transferred to the new YPD or MRS medium for continuous growing. Finally, the growth probiotics were harvested and dried by a fluidized bed. The free non-encapsulated cultured bacteria were harvested and dried by the same method, as a control.

### Scanning electron microscopy of microcapsules

Scanning electronic microscopy (SEM) was used to study the structure of microbial-loaded microcapsules before and after culture, according to a method previously described (Qi et al. 2005). The brief process was as follows: the microcapsules were embedded in paraffin, de-paraffin, and sputter-coated with gold. External regions of the microcapsules were observed using a scanning electron microscope (S-3000 N, Hitachi, Japan).

### Measurement of microcapsule diameter

Microcapsules (30 g), before and after culture, were dispersed in water, and a laser particle analyzer (Mastersizer-2000, Malvern Instruments, Ltd., Malvern, UK) was used to measure the diameters. Particle diameter distribution was reported using the volume moment mean diameter (D[4,3]), and each sample was analyzed in triplicate.

### Growth profiling

The same number of free and microencapsulated microbial cells was inoculated in sterile YPD or MRS broth in triplicate. Cell density was determined by measuring the optical density (OD) every 2 h over a 24-h time course. The ODs of bacteria-free broth and empty microcapsules were used as controls. Before the OD was measured, the microencapsulated probiotics were broken up using a chemical method (Xue et al. 2004), and the OD value was determined at 600 nm using an ultraviolet spectrometer (Lambda 35, PerkinElmer, USA).

### Heat and humidity tolerance

The heat tolerance of dry, free and microencapsulated, probiotics was studied by exposing 1 g samples to 110 °C or 130 °C each for 30 s, 45 s, or 60 s, in a heating and drying oven (9071A, Shanghai Jing Hong Laboratory Instrument Co., Ltd. China), and the untreated sample was used as a control. The tolerance of free and microencapsulated probiotics under both heat and humidity was assessed as follows: 1 g samples were exposed to 75 °C or 85 °C, each for 1 min at 100% humidity, determined by a hygrometer. After treatment, the survival rate of the probiotics was determined by subsequent serial dilutions and inoculation on YPD or MRS agar plates. Plates were incubated at 30 °C or 37 °C for 48 h. For enumeration of the microencapsulated probiotics, the bacteria were released from the capsules. All treatments were performed three times.

### Survival in simulated gastric conditions

Samples (0.5 g) of dry, free and microencapsulated, probiotics were added to tubes containing 4.5 mL of pre-warmed (37 °C) and filter-sterilized simulated gastric juice (SGJ) based on a modified method (Wei et al. 2011). The SGJ consisted of 0.02 M phosphate-buffered solution (PBS) with 10 mg mL^−1^ pepsin (P7000, Sigma), and pH was adjusted to 3.0 using 1 M HCl. The samples were incubated at 37 °C for 0, 30, 90, or 180 min at 80 rpm. After incubation, the splitting solution was added to neutralize the pH and release bacteria from the capsules, and the survivors were enumerated as described above. The challenge experiments were performed with three samples.

### Survival in simulated intestinal conditions

Survival of dry, free and microencapsulated, probiotics in simulated intestinal juice (SIJ) were carried out according to a modified method (Wei et al. 2011). Briefly, 0.5 g samples were added to tubes containing 4.5 mL pre-warmed (37 °C) filter-sterilized SIJ. The SGJ consisted of 0.02 M phosphate-buffered solution (PBS) with 10 mg mL^−1^ trypsin (T7409, Sigma), 0.3% bile salt (8008-63-7, Sinopharm Co., Ltd, China), and pH was adjusted to 6.8 using 1 M NaOH. The samples were incubated at 37 °C for 0, 30, 90, or 180 min at 80 rpm. The survivors were enumerated as described above. Each experiment was performed three times.

### Statistical analysis

Data were compared by a one-way analysis of variance and a Student’s *t* test. The results are expressed as mean ± standard deviation. Differences were considered significant at *p* < 0.05.

## Results

### Morphology of micro-beads before and after bacterial growth

**Fig. 1:**
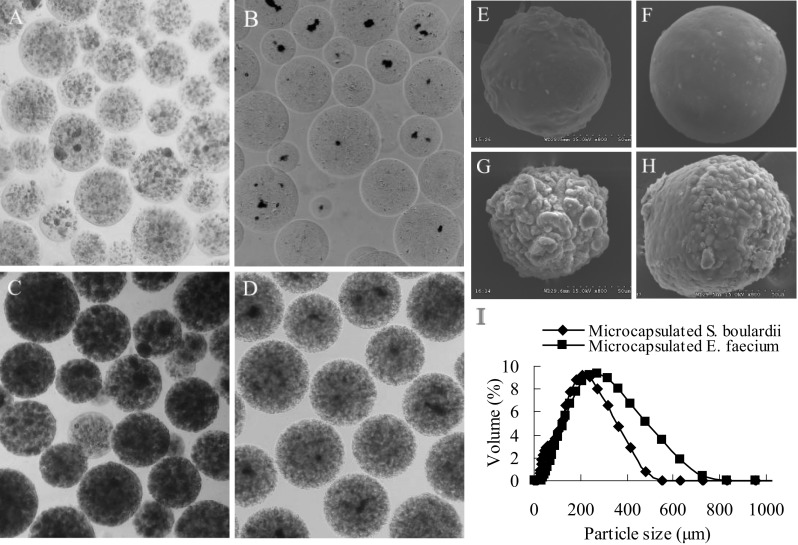
The morphology of micro-beads loaded with bacteria and their growth profile. **a**, **b** Micro images of freshly prepared micro-beads loaded with *S. boulardii* or *E. faecium*. **c** Micro-beads loaded with *S. boulardii* after 18 h of incubation in YPD broth with 180-rpm shaking at 30 °C. **d** Micro-beads loaded with *E. faecium* after 14 h of incubation in MRS broth at 37 °C. **e**, **f** Scanning electron micrograph of micro-beads freshly loaded with *S. boulardii* or *E. faecium*. **g**, **h** Scanning electron micrograph of micro-beads loaded with *S. boulardii* or *E. faecium*, after incubation. **i** The size distribution of micro-beads loaded with *S. boulardii* or *E. faecium* was measured by a laser particle analyzer (n = 3). All micro images show 40 × magnification

Figure 1 shows the morphology of the micro-beads loaded with *S. boulardii* and *E. faecium*, as well as their growth profiles. The micro-beads prepared by emulsion and internal gelation showed an intact, spherical, and uniformed appearance (Fig. 1a–d). Results from SEM showed that the surface of the micro-beads were spherical and smooth when freshly prepared, while the wrinkled surface was observed after incubation (Fig. 1e–h).

Figure 1I shows the size distribution of micro-beads measured by a laser particle analyzer. The mean particle size of the microcapsules loaded with *S. boulardii* and *E. faecium* was about 305 and 425 μm, respectively.

### Growth profiles of microbe cells in micro-beads

**Fig. 2:**
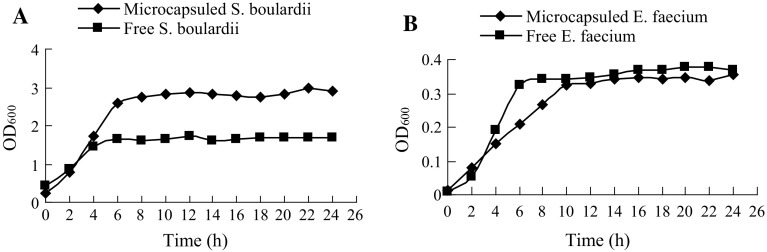
The growth profiles of micro-beads loaded with bacteria were investigated by measuring the cell density as a function of time. **a** The growth curve of free and microencapsulated *S. boulardii*, with the cell density measured every 2 h (n = 3). **b** The growth curve of free and microencapsulated *E. faecium*, with the cell density measured every 2 h (n = 3)

The growth profiles of *S. boulardii* and *E. faecium* in microcapsules compared to free culture were investigated by measuring the cell density as a function of time. *S. boulardii* in microcapsules and free culture reached a maximum cell density at about 7 h and 6 h, respectively (Fig. 2a). A significant difference in cell growth rate between the two conditions was not found, but the maximum cell optical density of microencapsulated *S. boulardii* was about 1.74 times higher than that of the free culture. A lower growth rate was investigated under microencapsulated culture of *E. faecium* compared to the free culture. However, the maximum cell density exhibited no significant difference between the free and microencapsulated cultures.

### Heat and humidity tolerance

The survival rates of both *S. boulardii* and *E. faecium* were significantly decreased by high temperature and humidity. The survival rate of *S. boulardii* was 58.56 ± 2.81% and 57.83 ± 1.11% at 75 °C and 85 °C, respectively, with 100% humidity when the cells were non-encapsulated which means 41.44% and 42.17% decrease at 75 °C and 85 °C, respectively. The survival rate were 87.92 ± 4.15% and 83.54 ± 3.16%, and the decreases were 12.08% and 16.46% when the microbial cells were microencapsulated, indicating about a 25% improvement in *S. boulardii* survival rate under both temperatures. The survival rate of *E. faecium* was 41.76 ± 3.12% and 37.63 ± 1.87% at 75 °C and 85 °C, respectively, with 100% humidity under non-encapsulated conditions, while the data were 88.50 ± 2.84% and 72.78 ± 2.67% respectively under encapsulated conditions. The improvements were 47% and 35% at 75 °C and 85 °C respectively, which means a more significant protection by microencapsulation was found when *E. faecium* was treated.

Free and microencapsulated probiotics were placed at 110 °C or 130 °C, each for 30 s, 45 s, or 60 s to further evaluate the protection of microencapsulation at high temperature. The results showed a decrease in the survival rate with increased temperature and treatment time, especially for *E. faecium* (Table 1). The differences between free and microencapsulated microbes were significant (*p* < 0.05), especially at 130 °C. There was a 9–22% increase in the survival rate of *S. boulardii* under microencapsulated conditions compared to free *S. boulardii*. Free *E. faecium* can barely survive these harsh conditions, but the survival rate reached 50–80%, or showed increases of 42–80%, when microencapsulated.

**Table 1:** The survival rate of free and microencapsulated bacteria under the conditions of high temperature (%)

Temperature	Time (s)	*S. boulardii*		*E. faecium*	
		Free	Microencapsulated	Free	Microencapsulated
110 °C	30	90.98 ± 1.06^a^	96.04 ± 1.30^b^	4.94 ± 0.25^a^	85.30 ± 5.46^b^
	45	90.26 ± 2.55^a^	95.11 ± 2.73^a^	4.84 ± 0.18^a^	68.42 ± 2.48^b^
	60	88.46 ± 3.49^a^	92.19 ± 2.98^a^	1.84 ± 0.09^a^	43.54 ± 2.20^b^
130 °C	30	82.70 ± 1.30^a^	92.50 ± 1.74^b^	0.60 ± 0.03^a^	65.49 ± 0.55^b^
	45	77.46 ± 1.74^a^	91.25 ± 2.19^b^	0.52 ± 0.02^a^	61.79 ± 0.65^b^
	60	70.12 ± 2.00^a^	87.29 ± 4.38^b^	0.40 ± 0 .03^a^	57.37 ± 0.65^b^

^a,b^Means within a row with no common superscript differ significantly (*p* < 0.05). Identical small letter superscripts indicate no significant difference (*p* > 0.05) (n = 3)

### Survival in simulated gastric and intestinal conditions

SGJ led to a significant decrease of survival rate of both *S. boulardii* and *E. faecium.* The survival rate of free *S. boulardii* cells was only 29.19%, 23.85%, and 18.61% when the cells were treated for 30, 90, and 180 min, respectively (Table 2). The survival rate of microencapsulated *S. boulardii* was 89.62%, 82.86%, and 74.53%, which indicates 60%, 59%, and 56% increases in cell survival under SGJ conditions. Survival improvements of 24%, 29%, and 26% were detected when *E. faecium* was examined (Table 2). The same results were observed in SIJ conditions. The survival rate of *S. boulardii* cells was improved by 16%, 15%, and 25% in microencapsulated conditions when the SIJ treatment time was 30, 90, and 180 min, respectively, and the percentages of improvement were 19%, 18%, and 19% for *E. faecium* (Table 2).

**Table 2:** The survival rate of free and microencapsulated bacteria in the condition of SGJ and SIJ (%)

Simulated conditions	Time (min)	*S. boulardii*		*E. faecium*	
		Free	Microencapsulated	Free	Microencapsulated
SGJ	30	29.19 ± 2.61^a^	89.62 ± 4.25^b^	58.91 ± 1.36^a^	83.70 ± 2.48^b^
	90	23.85 ± 1.17^a^	83.86 ± 4.29^b^	49.07 ± 0.28^a^	78.02 ± 4.67^b^
	180	18.61 ± 2.49^a^	74.53 ± 4.11^b^	38.27 ± 0.52^a^	64.78 ± 2.19^b^
SIJ	30	80.61 ± 2.23^a^	94.48 ± 2.52^b^	59.98 ± 3.06^a^	79.31 ± 3.56^b^
	90	65.42 ± 2.16^a^	82.14 ± 1.90^b^	50.26 ± 0.53^a^	68.88 ± 3.56^b^
	180	41.68 ± 2.05^a^	67.53 ± 2.39^b^	37.04 ± 1.37^a^	55.77 ± 7.24^b^

^a,b^Means within a row with no common superscript differ significantly (*p* < 0.05). Identical small letter superscripts indicate no significant difference (*p* > 0.05) (n = 3)

## Discussion

For the last three decades, microencapsulation has been investigated as a means to protect probiotics cells from the negative influences of in vitro and in vivo conditions. One scalable and low-shear alternative for generating alginate beads is emulsion and internal gelation. In this paper, *S. boulardii* and *E. faecium* were microencapsulated by the emulsion and internal gelation method. The survivability of the microencapsulated probiotics in adverse conditions was further evaluated.

The micro-beads prepared by this method showed an intact, spherical, and uniform appearance during the entire culture process, with shaking at 180 rpm, for both *S. boulardii* (Fig. 1a, c) and *E. faecium* (Fig. 1b, d). To further investigate the morphological characteristics of the micro-beads, SEM was used. A wrinkled surface was observed during proliferation of the microbe cells, but the micro-beads under both conditions were unbroken, even though slight expansion was seen when the microcapsules were loaded with *S. boulardii* (Fig. 1h). Usually, a membrane is formed by some compounds, such as polylysine and chitosan, around the alginate micro-beads for better protection of the cells and prevention of cell leakage, especially when the microcapsules are used to protect transplanted cells from attack by the host immune system (Qi et al. 2006; Chan et al. 2014). Sometimes the microcapsule core is liquefied to offer more space for cell proliferation (Qi et al. 2005; Kim et al. 2014). Both core liquefaction and membrane formation were omitted in this research, but we did not find obvious leakage of the microbe cells. Moreover, the bacteria grew well and reached a very high density in the micro-beads (Fig. 1c, d). This modification would be very important for lower cost and simplification of the process when microencapsulated products are used as food or feed supplements.

The size distribution of micro-beads prepared in this study was about 300–500 μm. It has been shown that there is a suitable size for microbe microencapsulation (Qi et al. 2005). The particle size not only affects the stability of microcapsules, but also influences the mass transfer for cell growth and metabolism. Larger beads offers better protection and larger spaces to cells than smaller ones, though mass transfer to the interior of the cell aggregates can be limited even though more cell aggregates are formed, especially in the center of the micro-beads (Qi et al. 2005). However, too big size of diameter will lead to easier broken of micro-beads by the shear force during the stirring and culture process. As a food additive of encapsulated probiotics, a particle size range of 50–500 μm is recommended (Chitprasert et al. 2012). Thus, the emulsion and internal gelation method proposed in this paper can produce microcapsules of appropriate size for microbe cell envelopment and proliferation.

The maximum cell optical density of microencapsulated *S. boulardii* was significantly higher than that of the free culture. A possible reason is that the micro-beads can provide a microenvironment with low shear stress. Moreover, microcapsules promoted cell aggregation, which in turn enhanced the microbe density. A lower growth rate was observed under encapsulated culture of *E. faecium*. We presumed that the stress resistance of *E. faecium* was lower than that of *S. boulardii*, and a low activity of *E. faecium* was caused by the process of micro-beads preparation. The encapsulated cells need more time than free ones to fit the conditions. However, the maximum cell density exhibited no significant difference between free and microencapsulated cultures. The growth profiles further prove that the micro-beads, without a membrane or liquefied core, offer enough protection and guarantee mass transfer for microbial cell proliferation.

As function food or medicine, Probiotics are usually processed before used by human being, in which high temperature and humidity are needed. The survival of microbial cells is critical for probiotics to maintain their function. In this study, we evaluated the survival of free and microencapsulated probiotics at high temperature and humidity. The survival rates of both *S. boulardii* and *E. faecium* were significantly decreased by high temperature and humidity, while these decreases were significantly reduced when the microbial cells were microencapsulated. Similar results have been reported, showing that microencapsulated bacteria had enhanced survival compared to free bacteria, with an average loss of only 4.17-log cfu mL^−1^ at 65 °C for up to 1 h (Ding and Shah 2007). Thus, microencapsulation can be considered a promising method for the protection of bacteria sensitive to high temperature.

We next compared the survival rate of microencapsulated probiotics with that of free ones under simulated in vivo conditions using gastric (SGJ) and intestinal juice (SIJ). SGJ and SIJ led to significant decreases in the survival rates of both *S. boulardii* and *E. faecium,* while the survival rates of microencapsulated cells were significantly improved. Usually, the more microbial cells that survive gastric and intestinal conditions, the better functions the probiotics bring to the host. However, most exogenous microbial cells can hardly retain activity in the low pH of the stomach or in the complex multi-enzyme conditions of the intestine (Iyer et al. 2004). *S. boulardii* and *E. faecium* are probiotics that are currently widely used in the food or even medicine industry. Results from this study show that neither of the two microbial cells under non-encapsulated conditions survives well in gastric and intestinal conditions, while the viable counts of microencapsulated ones show a significant increase. This indicates that alginate microencapsulation of microbial cells offers protection against in vivo conditions.

## Conclusion

This study demonstrates the feasibility of modified emulsion and internal gelation when applied to microencapsulation of probiotics. The prepared micro-beads exhibited a suitable size and morphological structure for the growth and metabolism of microbial cells. The results indicate good protection of probiotics at high temperature and humidity, as well as SGJ and SIJ conditions, by microencapsulation. With its low cost and simple process, emulsion and internal gelation is suitable for large-scale production and use of microcapsules in the food and medicine industry, and potentially for oral delivery of probiotics to the intestines.

## References

[CR1] Anal AK, Harjinder S (2007). Recent advances in microencapsulation of probiotics for industrial applications and targeted delivery. Trends Food Sci Technol.

[CR2] Arihara K (2006). Strategies for designing novel functional meat products. Meat Sci.

[CR3] Ashraf R, Shah NP (2014). Immune system stimulation by probiotic microorganisms. Crit Rev Food Sci Nutr.

[CR5] Chan KW, Liu G, van Zij PC, Bulte JW, McMahon MT (2014). Magnetization transfer contrast MRI for non-invasive assessment of innate and adaptive immuneresponses against alginate-encapsulated cells. Biomaterials.

[CR6] Chitprasert P, Sudsai P, Rodklongtan A (2012). Aluminum carboxymethyl cellulose–rice bran microcapsules: enhancing survival of *Lactobacillus reuteri* KUB-AC5. Carbohydr Polym.

[CR7] Ding WK, Shah NP (2007). Acid, bile, and heat tolerance of free and microencapsulated probiotic bacteria. J Food Sci.

[CR8] Gerez CL, Font de Valdez G, Gigante ML, Grosso CRF (2012). Whey protein coating bead improves the survival of the probiotic *Lactobacillus rhamnosus* CRL 1505 to low pH. Lett Appl Microbiol.

[CR9] Gill EE, Franco OL, Hancock RE (2015). Antibiotic adjuvants: diverse strategies for controlling drug-resistant pathogens. Chem Biol Drug Des.

[CR10] Gismondo MR, Drago L, Lombardi A (1999). Review of probiotics available to modify gastrointestinal flora. Int J Antimicrobial Ag.

[CR11] Guan HN, Chi DF, Yu J, Li H (2011). Encapsulated ecdysone by internal gelation of alginate microspheres for controlling its release and photostability. Chem Eng J.

[CR12] Hempel S, Newberry S, Ruelaz A, Wang Z, Miles JN, Suttorp MJ, Johnsen B, Shanman R, Slusser W, Fu N, Smith A, Roth B, Polak J, Motala A, Perry T, Shekelle PG (2011). Safety of probiotics used to reduce risk and prevent or treat disease. Evid Rep Technol Assess (Full Rep).

[CR13] Hill C, Guarner F, Reid G, Gibson GR, Merenstein DJ, Pot B, Morelli L, Canani RB, Flint HJ, Salminen S (2014). Expert consensus document. The international scientific association for probiotics and prebiotics consensus statement on the scope and appropriate use of the term probiotic. Nat Rev Gastroenterol Hepatol.

[CR14] Hoesli CA, Raghuram K, King RLJ, Mocinecová D, Hu XK, Johnson JD, Lacík I, Kieffer TJ, Piret JM (2011). Pancreatic cell immobilization in alginate beads produced by emulsion and internal gelation. Biotechnol Bioeng.

[CR16] Iyer C, Kailasapathy K, Peiris P (2004). Evaluation of survival and release of encapsulated bacteria in ex vivo porcine gastrointestinal contents using a green fluorescent protein gene-labelled *E. coli*. Food Sci Technol.

[CR34] John BW, Paul MC, Robert MR, Johanna TD, James AS, Barbara AB, Sarah AP (2011). Economic analysis of nutrition interventions for chronic disease prevention: methods, research, and policy. Nutr Rev.

[CR17] Kim BJ, Park T, Moon HC, Park SY, Hong D, Ko EH, Kim JY, Hong JW, Han SW, Kim YG, Choi IS (2014). Cytoprotective alginate/polydopamine core/shell microcapsules in microbial encapsulation. Angew Chem Int Ed Engl.

[CR18] Krasaekoopt W, Bhandari B, Deeth H (2003). Evaluation of encapsulation techniques of probiotics for yoghurt. Int Dairy J.

[CR19] Lee KY, Heo TR (2000). Survival of *Bifidobacterium longum* immobilized in calcium alginate beads in simulated gastric juices and bile salt solution. Appl Environ Microbiol.

[CR20] Mattila ST, Myllärinen P, Crittenden R, Mogensen G, Fondén R, Saarela M (2002). Technological challenges for future probiotic foods. Int Dairy J.

[CR21] Paulina M, Katarzyna Ś (2017). Effects of probiotics, prebiotics, and synbiotics on human health. Nutrients.

[CR22] Poncelet D, Poncelet De Smet B, Beaulieu C, Huguet ML, Fournier A, Neufeld RJ (1995). Production of alginate beads by emulsification/internal gelation. II. Physicochemistry. Appl Microbiol Biotechnol.

[CR23] Qi WT, Yu WT, Xie YB, Ma XJ (2005). Optimization of *Saccharomyces cerevisiae* culture in alginate–chitosan–alginate microcapsule. Biochem Eng J.

[CR24] Qi WT, Ma J, Yu WT, Xie YB, Wang W, Ma XJ (2006). Behavior of microbial growth and metabolism in ACA microcapsules and its modeling. Enzyme Microb Technol.

[CR25] Rokka S, Rantamäki P (2010). Protecting probiotic bacteria by microencapsulation: challenges for industrial applications. Eur Food Res Technol.

[CR26] Saarela M, Mogensen G, Fondén R, Mättö J, Mattila-Sandholm T (2000). Probiotic bacteria: safety, functional and technological properties. J Biotechnol.

[CR27] Vaithilingam V, Quayum N, Joglekar MV, Jensen J, Hardikar AA, Oberholzer J, Guillemin GJ, Tuch BE (2011). Effect of alginate encapsulation on the cellular transcriptome of human islets. Biomaterials.

[CR28] Vandenplas Y, De Greef E, Devreker T, Veereman-Wauters G, Hauser B (2013). Probiotics and prebiotics in infants and children. Curr Infect Dis Rep.

[CR29] Wei K, Han W, Cheng MJ, Li AK, Zhang XL, Zhou TB, Jiang JJ (2011). Isolation and microencapsulation of *Lactococcus lactiss* from fowls: effects on broiler growth performance. Chin J Anim Nutr.

[CR31] Xue WM, Yu WT, Liu XD, He X, Wang W, Ma XJ (2004). Chemical method of breaking the cell-loaded sodium alginate/chitosan mocrocapsules. Chem J Chin U.

[CR32] Zhang L, Li J, Yun TT, Li AK, Qi WT, Liang XX, Wan YW (2015). Evaluation of pilot-scale microencapsulation of probiotics and product effect on broilers. J Anim Sci.

[CR33] Zhang L, Li J, Yun TT, Qi WT, Liang XX, Wang YW, Li AK (2015). Effects of pre-encapsulated and pro-encapsulated *Enterococcus faecalis* on growth performance, blood characteristics, and cecal microflora in broiler chickens. Poult Sci.

